# Prophylactic splenic artery embolization using n-butyl-2-cyanoacrylate and coils prior to endoscopic necrosectomy in a patient with necrotizing pancreatitis: A case report

**DOI:** 10.1016/j.radcr.2024.05.027

**Published:** 2024-06-01

**Authors:** Hiroki Kamada, Sota Oguro, Tatsuro Fukushi, Hiromitsu Tannai, Hideki Ota, Kei Takase

**Affiliations:** Department of Diagnostic Radiology, Tohoku University Hospital, Sendai, Japan

**Keywords:** Prophylactic embolization, NBCA (n-butyl-2-cyanoarylate), Polytetrafluoroethylene (PTFE)-coated microcatheter, Necrotizing pancreatitis, Endoscopic necrosectomy

## Abstract

We present a case of prophylactic endovascular embolization in a 51-year-old man with necrotizing pancreatitis (NP) before undergoing endoscopic necrosectomy (EN). Contrast-enhanced CT imaging revealed the presence of a walled-off necrosis (WON) surrounding the pancreas, with the splenic artery coursing through the cavity. The splenic artery was embolized using n-butyl-2-cyanoacrylate (NBCA) and coils to mitigate the risk of massive bleeding in EN. A newly developed polytetrafluoroethylene (PTFE)-coated microcatheter was used to inject NBCA, enabling embolization of a long segment of the splenic artery without adhering to the vessel wall. Coils were placed distal and proximal to the embolized segment to optimize control. Over 5 sessions of EN, no massive bleeding was encountered. This report demonstrates the benefits of utilizing PTFE-coated microcatheters for enhanced safety and maneuverability during embolization with NBCA. Furthermore, it highlights the importance of prophylactic embolization during EN for managing NP.

## Introduction

Vascular injury is a critical complication of endoscopic necrosectomy (EN) in necrotizing pancreatitis (NP) [1]. Prophylactic radiological embolization has shown effectiveness in preventing intraoperative hemorrhage [2]. Metallic coils and n-butyl-2-cyanoacrylate (NBCA) are used for embolizing the pancreaticoduodenal and splenic arteries. NBCA, as a liquid embolization material, is quick-acting and suitable for embolizing long sections; however its behavior during injection is difficult to predict [3]. From our experience, handling NBCA during embolization of long segments can be challenging. This is due to potential migration to the periphery, reflux to the central side, and adherence to the catheter tip, leading to hesitation in its use. In this case, prophylactic splenic artery embolization with NBCA and coils was performed before initiating direct endoscopic necrosectomy (DEN). After dense embolization of the peripheral side with coils, a long segment was embolized with NBCA, and additional coils were placed on the central side, resulting in successful embolization. The use of a polytetrafluoroethylene (PTFE)-coated microcatheter enabled slow injection of NBCA and precise control over the range of embolization of the splenic artery inside the walled-off necrosis (WON).

## Case presentation

**Fig. 1:**
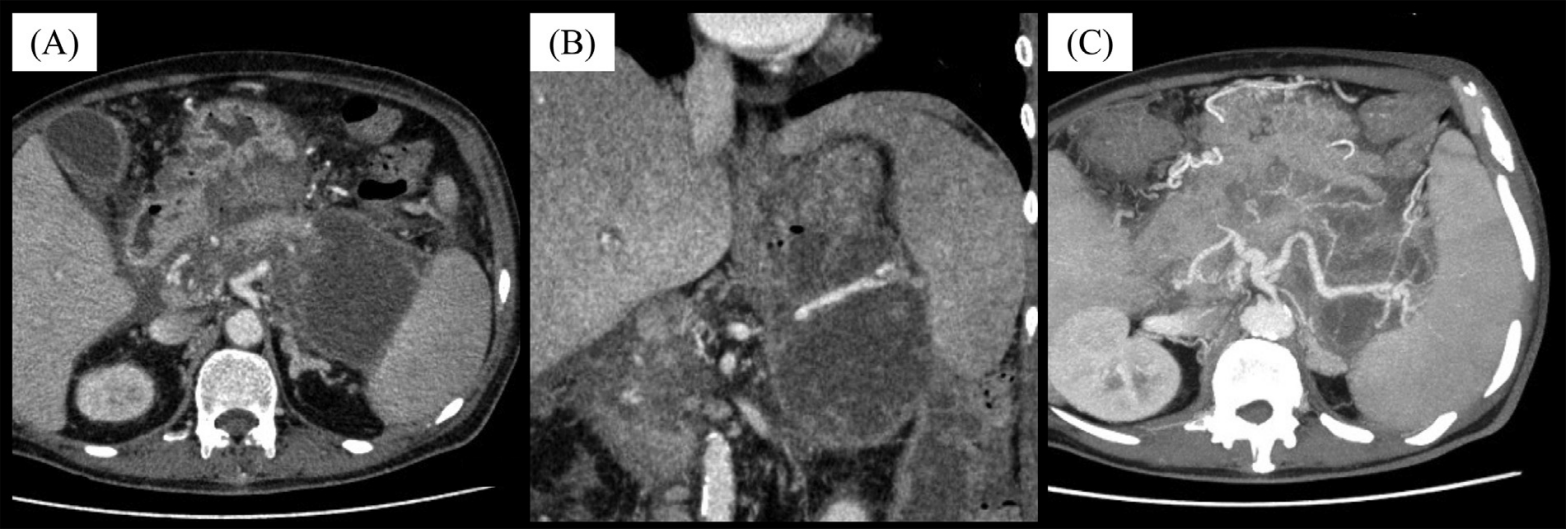
Contrast-enhanced CT at the time of transfer to our hospital. The walled-off necrosis (WON) is demonstrated around the pancreas in the (A)axial and (B) coronal views. The splenic artery is entirely distributed inside the WON in the (C) maximum intensity projection image.

A 51-year-old man with a history of chronic pancreatitis had been hospitalized for acute pancreatitis at a local general hospital for 4 months. A contrast-enhanced CT (CE-CT) scan revealed WON surrounding the pancreas. An endoscopic drainage of the WON was performed, but reduction of the WON was not achieved. Consequently, the patient was transferred to our hospital for DEN. A subsequent CE-CT scan upon transfer revealed a WON with multifocal cystic structures extending from the body to the tail of the pancreas; the main trunk of the splenic artery was coursing through the interior of the WON, accompanied by mild dilatation and irregularity in its caliber (Fig. 1). Given the risk of vascular injury to the splenic artery and its branches during DEN, it was deemed necessary to perform a prophylactic splenic artery embolization based on interventional radiology assessment.

**Fig. 2:**
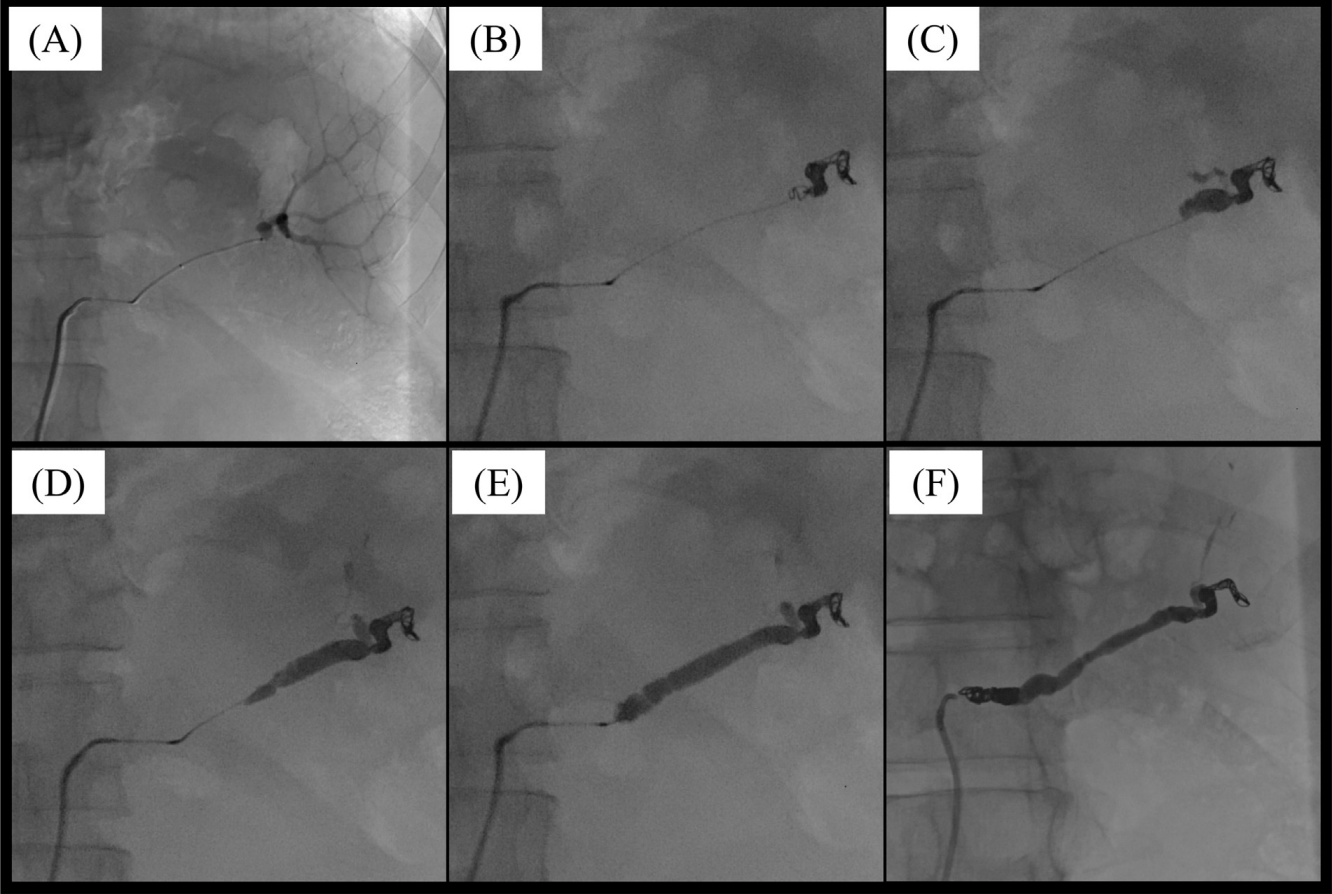
Prophylactic embolization of the splenic artery with n-butyl-2-cyanoacrylate (NBCA) and coils. (A) A triple coaxial system was advanced to the distal splenic artery. A mild arterial dilatation was suspected at the splenic hilum site. (B) Three coils were placed at the distal site of the splenic artery. (C-E) NBCA was slowly injected and embolized from proximal to the coil to the proximal site of the splenic artery. (F) Four detachable coils were added in the proximal site of the splenic artery.

The embolization was performed for the splenic artery. We decided to preserve the peripheral splenic artery on the splenic hilum side and embolize the long segment using NBCA in anticipation of collateral tract development from the short gastric and the left gastroepiploic arteries. A 6.5-Fr guiding sheath was inserted via the right femoral artery. A 4-Fr cobra-type catheter was inserted using a 0.035-inch guide wire, and the celiac artery was selected. A triple coaxial system with a 2.8/2.9-Fr (Coiling support EX SHK type, Hi-LEX corporation, Hyogo, Japan) and a 1.9-Fr (Carry Leon NSX, UTM, Aichi, Japan) microcatheters were inserted using a 0.016-inch microwire, and the splenic artery was selected. After advancing the system to the distal portion of the splenic artery (Fig. 2A), 2 detachable coils (Avenir, Japan Lifeline, Tokyo, Japan) and a pushable coil (C-stopper, Piolax Medical Devices, Kanagawa, Japan) were placed (Fig. 2B). After confirming that the contrast medium was stagnant in the splenic artery, an NBCA-lipiodol mixture (33% NBCA) was slowly injected from the coils to the proximal side of the splenic artery using the 1.9-Fr Carry Leon NSX microcatheter. Here, we repeated retracting a small amount of NBCA and pulling back the microcatheter for about 90 seconds (Figs. 2C-E). In addition, four detachable coils (Embold, Boston Scientific Japan, Tokyo, Japan) were placed from the Coiling support EX microcatheter (Fig. 2F). Post-embolization digital subtraction angiography (DSA) demonstrated the splenic arterial trunk occlusion.

**Fig. 3:**
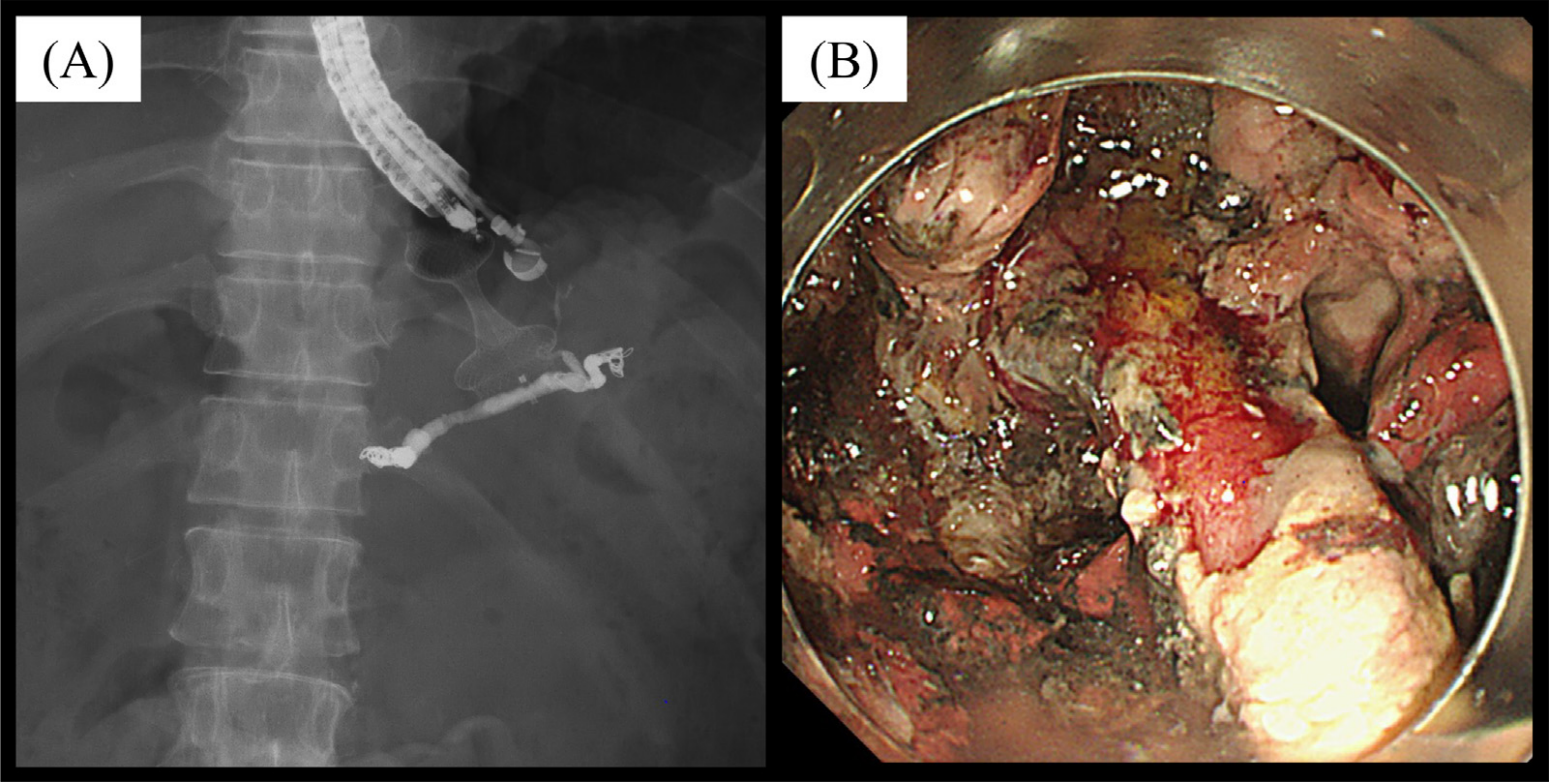
Direct endoscopic necrosectomy (DEN) was conducted after embolization. (A) Sessions of DEN were performed after a lumen-apposing metal stent was implanted. (B) Endoscopically, an embolized splenic artery was observed within the WON.

**Fig. 4:**
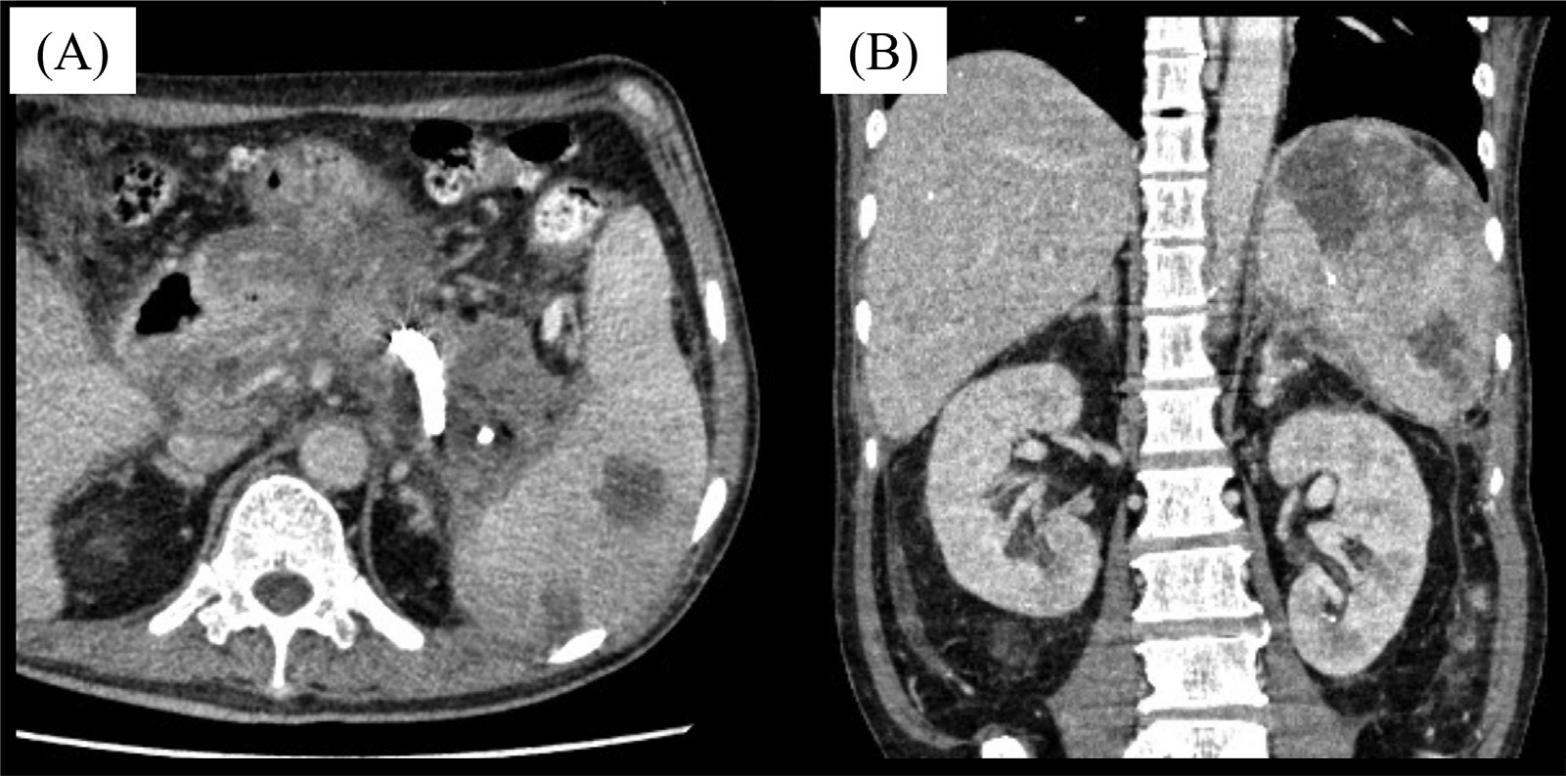
Contrast-enhanced CT after DEN treatment. (A) The WON around the pancreas has been reduced in size. (B) Embolic material remains in the splenic artery. Partial splenic infarction is seen.

A lumen-apposing metal stent (LAMS) placement was performed the day after embolization. The patient underwent 5 sessions of DEN without resulting in massive hemorrhage. Endoscopically, the main trunk of the splenic artery within the necrotic tissue was observed (Fig. 3). A CE-CT scan after DEN showed a reduction of the WON. The splenic infarction was confined to a narrow range of approximately 30% (Fig. 4). The patient was transferred to the local general hospital since his progress was good, with decreased pancreatic enzymes and inflammatory markers.

## Discussion

Prophylactic splenic artery embolization prior to DEN was successfully performed using a combination of NBCA and coils. NBCA has been reported to be effective because of its immediate effectiveness and ability to achieve embolization even in cases of reduced coagulability [3]. However, despite its effectiveness, NBCA has drawbacks such as challenges in predicting its behavior during injection and controlling the embolization area. Furthermore, NBCA's tendency to adhere to the catheter and vessel wall can reduce maneuverability and safety [3].

The Carry Leon NSX is a newly developed microcatheter with a PTFE-coated tip [4]. The PTFE coating offers safer injection of NBCA than conventional microcatheters, owing to its reduced adhesion to the NBCA. This microcatheter does not adhere to the arterial wall during NBCA infusion, eliminating the need for hasty removal of the microcatheter after infusion [4]. This feature of the microcatheter facilitates precise control of the embolic region. Embolizing a long segment of the splenic artery involves using numerous metallic coils, leading to prolonged procedure times. However, the use of NBCA reduced the number of coils and shortened the procedure time. No reports of clinical use of the Carry Leon NSX microcatheter have been confirmed. However, it is anticipated to enable safe NBCA injection in cases requiring arterial embolization of long segmental ranges, thereby contributing to shorter procedure times and cost reductions.

Several coils were placed distal to the splenic artery to prevent NBCA from flowing peripherally. Preserving the arterial branch of the splenic hilum region distal to the coils was considered to provide collateral blood flow to the spleen [5]. The splenic artery needed to be embolized proximally, as suggested by DSA before embolization. Detachable coils were used to embolize the proximal side of the splenic artery due to the need for tighter control of the embolic region to prevent deviation of the embolized material into the celiac artery. Consequently, the combined use of coils effectively controlled the embolization range of the NBCA.

Recently, the treatment of NP has shifted from surgical to endoscopic procedures. EN has been reported to have a high success rate [6]; however, vascular injury within the necrotic tissue remains a significant complication [7]. With the background of the fragility of the vascular wall due to proteolytic pancreatic enzymes [8], the vascular injury caused by external factors of EN would be more prone to occur. The frequency of vascular injury attributed to EN is estimated to be up to 20% [[9], [10], [11]], and fatalities due to bleeding have also been reported [12]. Therefore, prophylactic embolization is vital in cases where the gastric, pancreaticoduodenal, and splenic arteries are distributed within the WON cavity on preoperative imaging. In this case, massive bleeding did not occur during repeated DEN, suggesting that prophylactic embolization was effective. In the management of NP, a multidisciplinary approach involving gastroenterology as well as diagnostic and interventional radiology may improve the safety of DEN and contribute to improved outcomes and expanded indications.

## Conclusion

The splenic artery embolization with NBCA and coils was performed prior to DEN to prevent massive bleeding during the procedure. The utilization of a PTFE-coated microcatheter improved maneuverability and safety during NBCA injection and reduced procedure time. Moreover, the use of coils effectively controlled the embolization range.

## Patient consent

Written informed consent for the publication of this case report was obtained from the patient.
